# A Rare Case of Sacral Decubitus Injury Causing Methicillin-Resistant Staphylococcus aureus (MRSA) Bacteremia With a Spinal Abscess

**DOI:** 10.7759/cureus.110749

**Published:** 2026-06-12

**Authors:** Genevieve A Crawley, Suman Radhakrishna

**Affiliations:** 1 Internal Medicine, Ross University School of Medicine, Miramar, USA; 2 Infectious Disease, Ross University School of Medicine, Miramar, USA; 3 Surgery, Ross University School of Medicine, Miramar, USA; 4 Infectious Disease, Dignity Health - California Hospital Medical Center, Los Angeles, USA; 5 Infectious Disease, California Hospital Medical Center, Los Angeles, USA

**Keywords:** antibiotic administration in management of severe sepsis, antibiotic-resistant pathogens, bacterial infectious disease, infectious disease pathology, infectious-septic endocarditis, mrsa bacteremia, mrsa infective endocarditis, sacral decubitus ulcer, sacral pressure injury, severe sepsis

## Abstract

Sacral ulcers can be precipitated by poor circulation, damaged skin and tissue from immobility or direct trauma, poor nutrition, diabetes, and incontinence. If not treated promptly, the sacral ulcer can serve as a portal of entry for bacteria, such as methicillin-resistant *Staphylococcus aureus* (MRSA), into the bloodstream, leading to complications including bacteremia, abscesses, sepsis, and possible infectious endocarditis, as occurred in this case. During the patient's hospital stay, he was diagnosed with possible schizoaffective disorder with full mental capacity, but had multiple episodes of paranoia that made treatment planning even more difficult due to both his persistent refusal to diagnostic tests and physical therapy. The refusal of necessary diagnostic testing, particularly to rule out infectious endocarditis, limited treatment planning and could have provided important insight into his persistent bacteremia despite susceptible antibiotic therapy. Along with the sacral decubitus ulcer, the patient's case was complicated by paraspinal, psoas, epidural, and multiloculated gluteal abscesses with fungal infections around the groin. Overall, this case highlights the importance of correct antibiotic therapy, prevention, aggressive wound care, nutrition, and adherence to therapy recommendations, even if the patient has psychiatric issues, to mitigate severe complications associated with MRSA secondary to a sacral decubitus ulcer.

## Introduction

*Staphylococcus aureus* was first identified in 1871 by von Recklinghausen, and in 1880, the Scottish surgeon Ogston reported that the bacterium causes abscesses and suppurative diseases [1]. Later, methicillin was first introduced in 1959 as a treatment for penicillin-resistant *S. aureus* [2]. In the coming years, methicillin-resistant *S. aureus* (MRSA) was identified and became a problem among hospitals, nursing homes, and the community [3,4]. The acquired resistance of *S. aureus* to methicillin lies within its mecA gene that expresses penicillin-binding protein 2a to block any effects of β-lactam antibiotics such as methicillin [5,6]. This resistance to both methicillin and other antibiotics makes it even more challenging for the provider to establish a set treatment plan. This is one of the many problems that occurred in this patient's case. Along with this patient's MRSA being resistant to multiple treatments, the patient also had a sacral decubitus ulcer that acted as a portal of entry to allow MRSA into the body, then eventually into the bloodstream. Once in the bloodstream, the bacterium can spread to other organs throughout the body, causing complications such as abscesses, infective endocarditis, sepsis, and skin lesions. One of the factors that made this case particularly unique was the high clinical suspicion that the persistent source of infection originated within the heart itself, with infective endocarditis driving the dissemination of MRSA bacteremia to multiple organ systems. Due to this, it caused multiple abscesses, including spinal abscesses, sepsis, and skin lesions, throughout his body. However, the patient refused any confirmatory tests due to his psychological condition, which made it difficult to diagnose and treat.

## Case presentation

The patient is a 58-year-old febrile unhoused man with a past medical history of undiagnosed psychosis with paranoid ideation who came to the hospital for workup of abdominal pain, diarrhea, falls, and weakness. The patient was a poor historian with a Glasgow Coma Scale of 15 and noted to have paraplegia, a pressure injury to the left gluteal area with purulent drainage, and scabs at various stages of healing on extremities. The patient's vitals showed that he had slight tachycardia and was febrile, with all other lab values in the normal range (Table 1). Labs were drawn, which showed hyponatremia with a sodium of 123 (normal range: 135-145 mEq/L) and elevated blood sugar of 280 (fasting blood sugar in nondiabetic adult normal range: 70-99 mg/dL). Urine toxicology screening was not performed at admission. The patient’s hospital stay timeline and picture of his wounds are shown in Figures 1-3.

**Table 1:** Important lab values from beginning of hospital stay to toward end of hospital stay

Time	Sodium in blood (normal reference range: 135-145 mEq/L)	Fasting blood glucose in nondiabetic adult (normal reference range: 70-99 mg/dL)	Potassium (normal reference range: 3.5-5.2 mEq/L)
Beginning of hospital stay	123	280	3.1
Middle of hospital stay	128	280	3.1
Toward end of hospital stay	≈135	240	3.5

**Figure 1:**
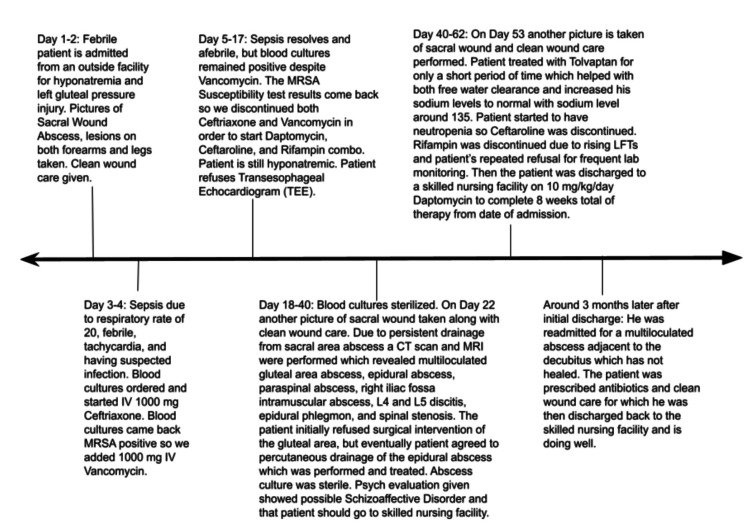
Hospital stay timeline of events from initial admission to three months later after initial discharge

**Figure 2:**
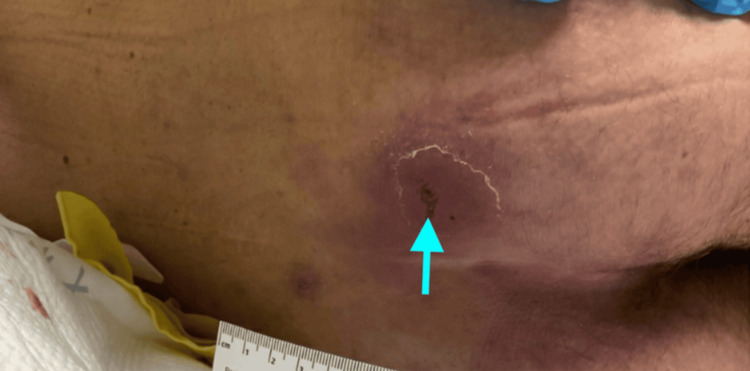
Day 1 picture of sacral wound abscess labeled by blue arrow

**Figure 3:**
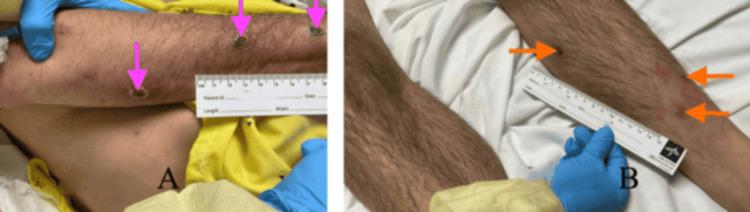
Healing skin lesions on Day 1. (A) Scabs on the right forearm labeled by pink arrow. (B) Scabs on bilateral lower legs labeled by orange arrow

The patient refused invasive procedures and surgical intervention. The patient was classified as a Stage 3 gluteal injury due to involving full-thickness skin loss with the fat layer exposed, but not bone, tendon, or muscle. This type of injury presents as a deep wound with rolled edges, healthy granulation tissue, or areas of dead tissue containing eschar and/or slough, and may include deep tissue pockets and wound extensions beneath the skin. This patient's Stage 3 gluteal injury was treated by clean wound care protocol including Medihoney, nutrition, and offloading. On Day 3, the patient developed sepsis with a respiratory rate of 20, febrile temperature of 102.2°F, and tachycardia, while having a suspected infection. On this same day, the first order of blood cultures was placed. Due to his sepsis, the patient was initially started on the standard 1,000 mg IV ceftriaxone; however, this antibiotic was not helping clear the infection. A common theme throughout the patient's hospitalization was that we would first start him with one antibiotic and then, over the course of a few days, the MRSA bacterium would gain resistance, so we were constantly switching antibiotics for much stronger ones. Continuing, on Day 4, the patient's blood cultures confirmed MRSA, for which 1,000 mg IV vancomycin was added to the antibiotic regimen until the susceptibility test results came back. Along with the patient's body weight, the dosage of vancomycin is given based on the patient's peaks (normal vancomycin peak range: 20-40 mcg/mL) and troughs (normal vancomycin trough range: 5-15 mcg/mL), with the goal of maintaining adequate therapeutic levels between those peaks and troughs. We also measure the patient's trough before administering their next dose of vancomycin. He continued to remain febrile and tachycardic, despite therapeutic levels of vancomycin administration. Serial blood cultures remained positive on Day 7, and testing was limited by his refusal to consent to any additional testing or surgery. Around this time, the results from the MRSA susceptibility test came back (see Table 2), which prompted us to switch both ceftriaxone and vancomycin to 500 mg IV daptomycin every 24 hours, 600 mg IV ceftaroline every 12 hours, and oral 300 mg rifampin twice daily. Blood cultures finally sterilized after 18 days of this new appropriate antibiotic treatment. Due to persistent drainage from the sacral area abscess, a CT scan was performed, which revealed a multiloculated gluteal area abscess and epidural abscess. The patient refused surgical intervention of the gluteal area, and local care was continued. Percutaneous drainage of the epidural abscess was performed on Day 40 of treatment. Abscess culture was sterile. It should also be noted that the patient had slowly arising fungal infections around the groin area that were being treated as well. He was noted to have neutropenia, which resolved after discontinuation of ceftaroline. Since the sodium in the blood was only minimally improving, it was decided to check his urine for any abnormalities. Along with this, it should be mentioned that the patient's urinary electrolytes started to appear like Syndrome of Inappropriate Antidiuretic Hormone Secretion (SIADH), in which the patient was put on tolvaptan for only a short period of time to help with both free water clearance and to increase his sodium levels to normal, with a sodium level around 135. Psych was consulted, and the patient was diagnosed with possible schizoaffective disorder with full mental capacity. Skilled nursing facility (SNF) placement was also recommended by psych due to the patient's inability to stand and ambulate. Finally, the patient allowed us to perform imaging around Day 34, which revealed multiple abscesses along with a paraspinal collection and epidural phlegmon. All blood cultures were negative and sterile. The progression of the patient’s sacral wound abscess is shown in Figure 4.

**Table 2:** Methicillin-resistant Staphylococcus aureus susceptibility/resistance chart for this patient (no change found from time of admission to discharge) N/A: not available; MINT: minimum inhibitory concentration

Drug	MINT (S = susceptible, R = resistant, I = intermediate)	Minimum detectable inhibitory level
Ampicillin	R	N/A
Cefazolin	R	N/A
Clindamycin	S	≤0.5
Doxycycline	S	≤0.5
Gentamicin	S	≤1
Oxacillin	R	>4
Rifampin	S	≤0.25
Tetracycline	I	8
Trimethoprim/sulfa	S	≤0.5/9.5
Vancomycin	S	1

**Figure 4:**
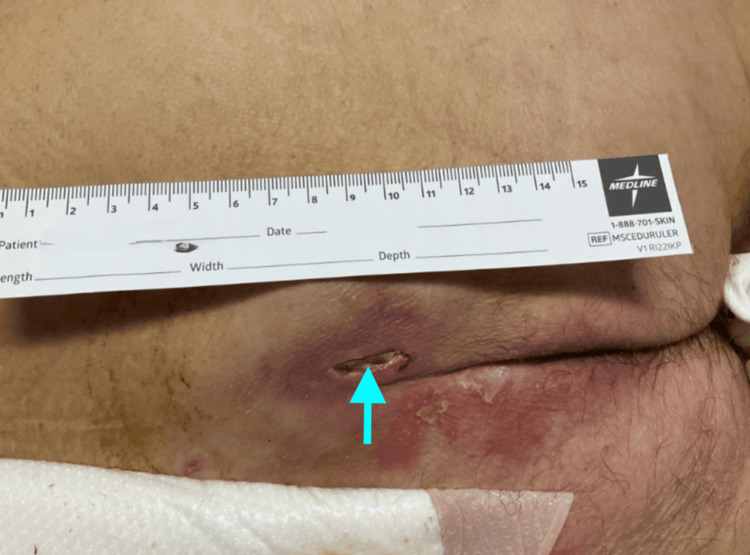
Day 22 picture of sacral wound abscess labeled by blue arrow

The CT scan of the pelvis with a paraspinal abscess and iliac fossa intramuscular abscess is shown in Figures 5-8.

**Figure 5:**
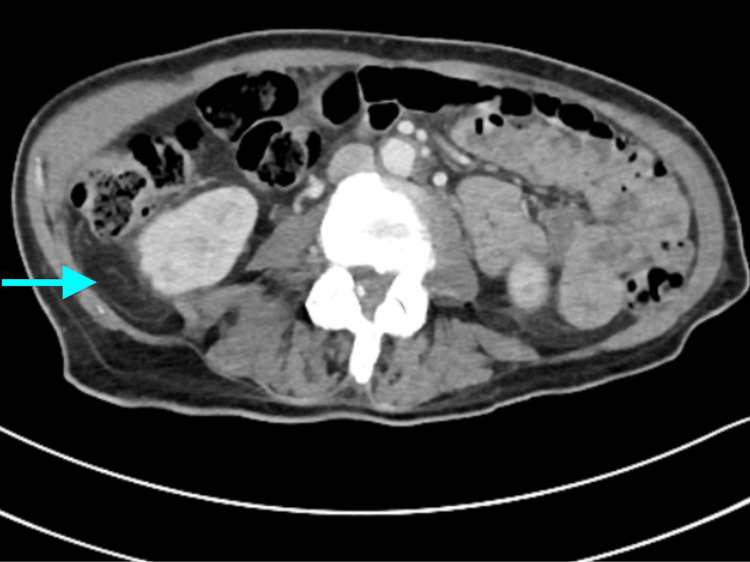
CT scan of the pelvis (axial view) with contrast showing a right iliac fossa intramuscular abscess labeled by blue arrow

**Figure 6:**
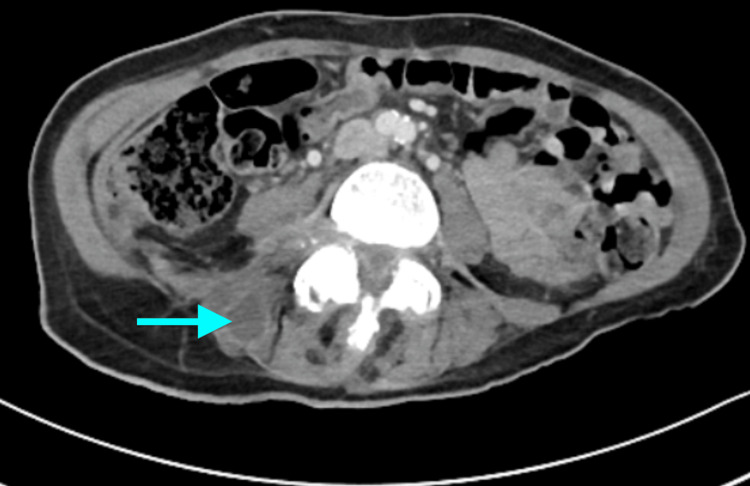
CT scan of the pelvis (axial view) with contrast showing a right paraspinal abscess labeled by blue arrow

**Figure 7:**
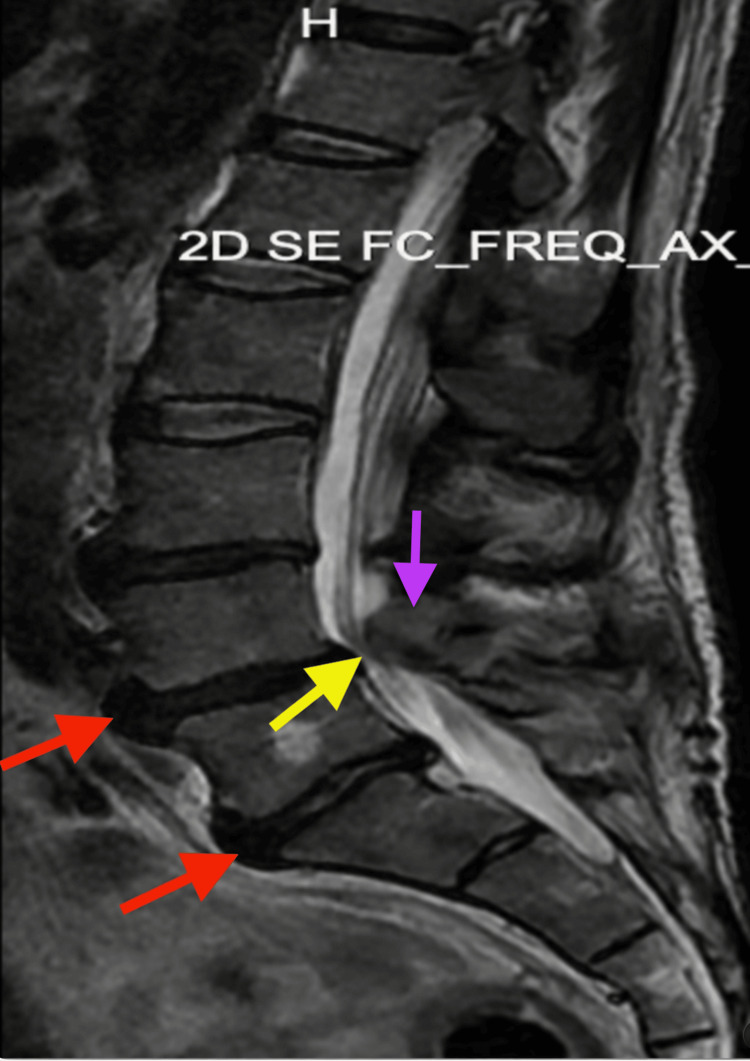
MRI of the lumbar spine with epidural abscess (sagittal view) containing L4 and L5 discitis both labeled by red arrow, epidural phlegmon labeled by purple arrow, and spinal stenosis labeled by yellow arrow

**Figure 8:**
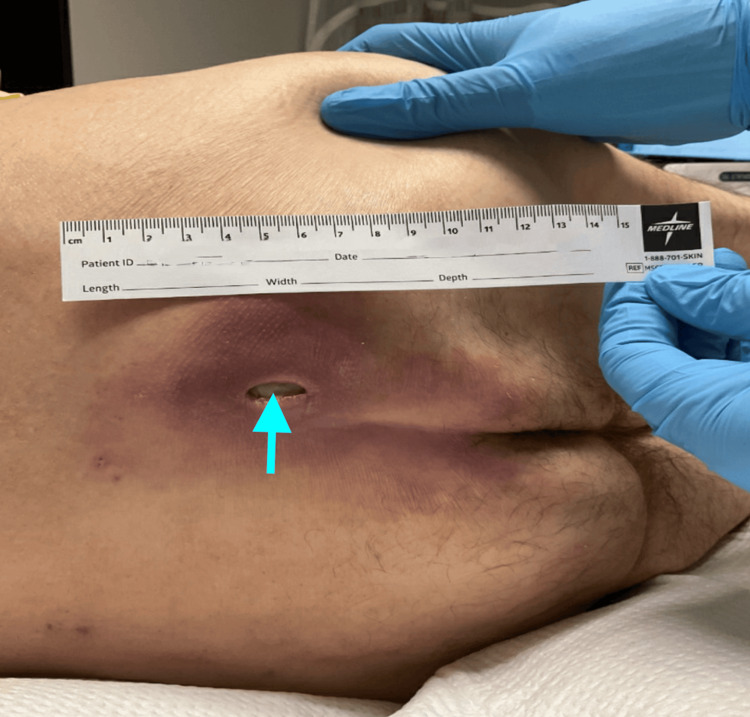
Day 53 picture of the sacral wound abscess around the time of discharge labeled by blue arrow

Rifampin was discontinued due to rising liver function tests and the patient’s repeated refusal for frequent lab monitoring. Then, the patient was discharged to an SNF on 10 mg/kg/day daptomycin to complete a total of eight weeks of therapy from the date of admission (the patient needed to be treated for at least six weeks from the date of negative cultures, since it was a resistant Gram-positive bacteremia). Three months after initial discharge, the patient was readmitted for a multiloculated abscess adjacent to the decubitus, which has not healed. The patient was prescribed antibiotics and clean wound care, after which he was discharged back to the SNF and is doing well.

## Discussion

Sacral decubitus ulcers are caused by a multitude of factors, including but not limited to poor circulation, immobility, and poor nutrition, which most likely occurred in this patient since he came to the hospital unhoused and with a psychological condition. Effective management of this patient's sacral decubitus ulcer requires source control and a comprehensive understanding of the extent of microbial dissemination, which was difficult to obtain due to his constant refusal of diagnostic tests, physical activity, and treatment recommendations. This patient’s case also drew attention to the other challenges of a sacral decubitus ulcer with MRSA bacteremia, such as multiple abscesses and possible infective endocarditis, which makes this case unique. Alarmingly enough, paraspinal abscesses can potentially cause paraplegia and/or affect urinary/bowel function via mass effect or vascular ischemia, in which the patient came in with apparent paraplegia, but luckily the other symptoms did not occur during his hospital stay. Continuing, due to this patient’s psychiatric issues, it was very difficult to obtain necessary diagnostic tests such as a transesophageal echocardiogram to definitively diagnose infectious endocarditis or any other possible source of infection, which made the treatment course limited to antibiotics and culture. In continuation, the patient's sacral decubitus ulcer acted as a portal of entry for the MRSA to enter the bloodstream, which eventually led to his persistent bacteremia.

Persistent MRSA bacteremia, defined as positive blood cultures for more than 1 week despite susceptible antibiotics, carries a high mortality rate that increases by 16% for each additional day the blood remains unsterile [7]. When adequate source control, such as removing infected catheters or draining abscesses, is not possible, management shifts to intensive medical salvage therapy, such as switching from standard monotherapy to high-dose polytherapy to combat antibiotic resistance [8]. It should also be noted that persistent MRSA bacteremia mortality rates are as high as 60%, which is why it is especially important to perform interventional radiology drainage and adequate antibiotic control promptly [9]. In relation to this, the patient in our case refused many tests and therapies due to his psychiatric condition, which made it difficult to drain and treat his abscesses and lesions. Also, a common theme throughout the patient's hospitalization was that we would first start with one antibiotic and then, over the course of a few days, his MRSA bacterium would gain resistance, so we were constantly switching antibiotics for much stronger ones. In this patient's case, he was initially started on IV ceftriaxone, but due to the intrinsic resistance patterns of his MRSA bacteremia, we had to switch him to vancomycin. Despite vancomycin, he continued to have persistent bacteremia secondary to poor source control since he would not let us perform the necessary tests. It should also be noted that abscesses naturally have an acidic pH, which likely contributed to vancomycin's limited efficacy in this patient due to its own acidic properties. We then deemed it a clinical failure and switched his therapy to IV daptomycin, ceftaroline, and rifampin combo, which eventually helped to sterilize the blood from MRSA. It should also be stated that rifampin can only be used once the blood culture is clear; otherwise, there is a potential for resistance as well. In general, with proper source control, the typical antibiotic duration is four to six weeks from the date of the first negative blood culture in patients with bone infection/osteomyelitis [9]. Even though the original source of this patient's bacteremia was unable to be found, this six-week-long treatment recommendation was applied since he had an atypical presentation of discitis/osteomyelitis.

In addition, it should be noted that, at the time of admission, the patient had hyponatremia, which is common among MRSA bacteremia patients who have sepsis. MRSA bacteremia is known to increase IL-6 inflammatory cytokines that stimulate antidiuretic hormone release, which will dilute sodium, thus causing an SIADH-related hyponatremia [10]. This information applies to this patient's case because his blood sodium levels were constantly low and urinary electrolytes appeared like SIADH despite certain treatments. His continuous chronic infection contributed to his SIADH symptoms. This is especially important knowledge for all physicians because antibiotics such as linezolid or tedizolid, sepsis-related kidney problems, or critical care fluid management can worsen already low sodium levels, thereby making hyponatremia an indicator of severe illness [10,11]. This is significant information because it helped us avoid using these antibiotics due to the patient's already hyponatremic state. We were able to demonstrate resolution of the infection at a later admission, which is often not feasible in these severe cases.

Finally, according to the CDC and the National Healthcare Safety Network, the United States has experienced a 16% decrease in hospital-onset MRSA bacteremia and has made overall progress in preventing several important healthcare-associated infections in acute care hospitals [12]. Simple precautions, such as good hand hygiene, help prevent transmission [12]. This is particularly important because the patient may have autoinoculated himself with the bacterium via rhinotillexis and/or dermatillomania from infected areas of his body to his sacral decubitus wound. Since this patient is psychiatric, this mode of transmission is more plausible, but again, the patient would not allow us to perform any necessary diagnostic tests, such as swabbing under his fingernails for possible microbes. This is why hand hygiene is crucial to prevent bacterial transmission [13].

## Conclusions

This case highlights the challenges of a sacral decubitus lesion, complexity of treating multiloculated and epidural abscesses, and how psychiatric comorbidities can affect the inability to achieve adequate source control along with management due to refusal of diagnostic and surgical interventions. In this patient patient's case prolonged combination antimicrobial therapy for MRSA bacteremia, multidisciplinary care, close clinical monitoring, wound management, nutritional support, and adaptation of treatment plans in the setting of limited patient cooperation was used. Also, one cannot disregard the possibility that the persistent bacteremia could have been a result of seeding of cardiac valves and subsequent infection of the lumbar spine/paraspinous area. For this reason, it is paramount to be aware of the clinical signs and symptoms of MRSA bacteremia, infectious endocarditis, and abscesses so that when an atypical presentation presentation arises that we are able to obtain a clinical diagnosis efficiently.
